# Ultrasonic Testing of Laser Welds in Medium-Thick Titanium Alloy Plates

**DOI:** 10.3390/s26072085

**Published:** 2026-03-27

**Authors:** Chenju Zhou, Jie Li, Shunmin Yang, Chenjun Hu, Kaiqiang Feng, Yi Bo

**Affiliations:** State Key Laboratory of Extreme Environment Optoelectronic Dynamic Measurement Technology and Instrument, North University of China, Taiyuan 030051, China; sz202306063@st.nuc.edu.cn (C.Z.); ysmsoft@nuc.edu.cn (S.Y.); hcj74839@gmail.com (C.H.); fkq0809@163.com (K.F.); sz202306068@st.nuc.edu.cn (Y.B.)

**Keywords:** titanium alloy, laser welding, ultrasonic testing, finite element method, defect localization

## Abstract

**Highlights:**

**What are the main findings?**

- For medium-thick titanium alloy laser welds, a shear wave inspection method was used to compare, via simulation, the acoustic field characteristics and defect responses at 1.5 MHz and 5 MHz. The simulation revealed that while the 1.5 MHz transducer yields a higher defect echo amplitude, its background response level increases correspondingly; conversely, the 5 MHz transducer exhibits a lower defect echo amplitude but a significantly reduced background response level. Both frequencies are capable of effectively detecting the defect in the model.
- The practical inspection capability of the 5 MHz transducer was validated experimentally. The results show that, while maintaining sufficient penetration for a 10 mm thickness, the 5 MHz transducer can effectively detect internal defects with a minimum equivalent size of 1 mm, and it achieves a localization accuracy better than 0.5 mm.

**What are the implications of the main findings?**

- This study addresses the ultrasonic inspection of the specific combination of titanium alloy, laser welding, and medium thickness. It provides a preliminary quantitative basis for transducer frequency selection by clarifying, through simulation and experiment, the trade-off between penetration depth and background response suppression.
- The integrated simulation–experimental approach validates the effectiveness of the established finite element model for studying ultrasonic propagation. It offers a valuable tool for optimizing inspection parameters and understanding the interaction between frequency, defect signal, and structural noise, reducing reliance on extensive physical trials.

**Abstract:**

To address the challenge of detecting internal defects in medium-thick titanium alloy laser welds, a combined simulation and experimental study on ultrasonic testing was conducted. A finite element model employing a 5 MHz shear wave angle transducer for inspecting titanium alloy welds was established. An ultrasonic testing system was developed, incorporating a DPR300 pulser-receiver (JSR Ultrasonics, Pittsford, NY, USA) and an MSO5204 oscilloscope (RIGOL, Suzhou, China), and was calibrated using standard reference blocks. The inspection results for four prefabricated internal defects at various depths demonstrated that all defects were effectively detected, with the minimum detectable equivalent defect size reaching 1 mm. The measured signal-to-noise ratio (SNR) averaged 17.6 dB, validating the high sensitivity of the proposed system. The mean absolute error for defect localization was 0.438 mm, achieving a positioning accuracy better than 0.5 mm. This study indicates that the pro-posed method enables effective detection and accurate localization of internal defects in titanium alloy laser welds, providing critical technical support for laser welding quality assessment.

## 1. Introduction

Titanium alloys have become key structural materials in fields such as shipbuilding, aerospace, and petrochemical engineering due to their high specific strength and excellent corrosion resistance [1,2]. However, the high material cost and difficulty of welding titanium alloys mean that the quality of welded joints critically determines the service reliability of high-end equipment. Laser welding offers advantages such as high energy density, minimal distortion, a narrow heat-affected zone, and the potential for large single-pass penetration. These characteristics make it a promising technique for joining medium-thick titanium alloy plates [3,4].

Medium-thick titanium alloy plates (typically with thickness ≥10 mm) are widely used in shipbuilding and marine engineering because of their excellent overall properties. They are employed in critical welded components such as submarine pressure hulls, deep-sea submersible crew cabins, seawater piping systems, heat exchangers, and propellers. The quality of these welds directly affects equipment safety and reliability. Currently, welding of such components primarily relies on TIG (Tungsten Inert Gas) and electron beam welding. The application of laser welding remains relatively immature. Existing research has largely focused on process optimization for thin TC4 titanium alloy sheets (<10 mm), with systematic studies on medium-thick plates being more limited [5,6].

Key challenges affecting joint quality during welding include plume suppression, surface oxidation control, and the prevention of porosity and cracks. Several researchers have undertaken exploratory improvements. For instance, Gao et al. demonstrated that high-quality welded joints free from lack-of-fusion defects could be achieved in thick titanium alloy plates using a multi-pass narrow-gap laser welding process [7]. Recent studies further indicate that ultrasonic vibration-assisted laser welding can refine weld grains and improve microstructure uniformity through cavitation and acoustic streaming effects, thereby enhancing the mechanical properties of the joint. Guo et al. found that ultrasonic vibration could induce a columnar-to-equiaxed grain transition in Ti6Al4V titanium alloy laser welds, resulting in a joint tensile strength of 945.2 MPa [8]. Nevertheless, the quality evaluation system for laser welds in medium-thick titanium alloy plates (≥10 mm) remains underdeveloped, particularly regarding the adaptability of non-destructive testing methods. Therefore, establishing effective means for weld quality assessment is of significant practical importance for improving welding processes and ensuring the reliability of high-end equipment.

Currently, the quality assessment of laser welds primarily relies on in-process monitoring of welding parameters and post-weld destructive testing. The former provides limited information and cannot accurately reflect internal weld quality, while the latter, although capable of obtaining geometric parameters such as penetration depth and bead width, is inefficient and costly [9,10]. Consequently, the development of non-destructive testing methods holds significant engineering value. Non-destructive testing (NDT) techniques are crucial means for ensuring the safety and reliability of welded structures. Common NDT methods include radiographic testing, ultrasonic testing, magnetic particle testing, penetrant testing, and eddy current testing, each with its specific application scope and technical characteristics. For instance, radiographic testing can be used to evaluate weld quality in thick-walled structures such as submarine pressure hulls and deep-sea submersible crew cabins, while penetrant testing or eddy current testing is often employed for surface and near-surface defect screening in thin-walled structures like seawater piping systems. Radiographic testing can visually reveal internal defects through imaging but has lower sensitivity to planar defects like cracks and involves radiation protection requirements. Magnetic particle and penetrant testing are suitable for detecting surface and near-surface defects but cannot detect internal flaws; moreover, penetrant testing requires a high surface finish. Eddy current testing is sensitive to surface and near-surface defects in conductive materials but has limited penetration depth and is significantly affected by variations in material magnetic permeability [11,12,13,14].

Ultrasonic testing, owing to its advantages such as strong penetrating power, high sensitivity, and harmlessness to the human body, is widely used for inspecting metal welds. However, compared to common carbon steel, titanium alloys have lower density and significantly different acoustic impedance, and their weld microstructures are characterized by coarse grains and anisotropy, leading to specific challenges in ultrasonic inspection. Research indicates that the main technical bottlenecks for ultrasonic testing of titanium alloy welds include: (1) High structural noise levels, resulting in a significantly lower signal-to-noise ratio (SNR) for defect echoes compared to carbon steel welds; (2) Enhanced sound scattering and attenuation caused by coarse grains, leading to sound velocity variations and increased errors in defect localization and sizing; (3) The weld reinforcement and surface irregularities further exacerbate the near-surface dead zone issue [15,16,17,18]. Currently, ultrasonic testing standards for titanium alloys are mostly oriented towards raw material acceptance. Inspection procedure specifications specifically for titanium alloy welds, particularly for medium-thick plates, are still inadequate. Consequently, the engineering applicability of these methods remains significantly limited compared to established inspection systems for materials like fine-grained ferritic steel [19,20].

To address the aforementioned technical bottlenecks in the ultrasonic inspection of medium-thick titanium alloy laser welds—namely high structural noise, low SNR, and difficulties in defect localization and sizing—this paper investigates a dedicated inspection procedure. Based on the analysis of ultrasonic propagation characteristics in titanium alloy welds, a dedicated small-element shear wave angle transducer was designed to suppress structural noise, improve the SNR of defect echoes, and extend the coverage of the weld inspection area. Calibration blocks, reference blocks suitable for inspecting medium-thick titanium alloy welds, and laser-welded test specimens (10 mm thick) were prepared. Subsequently, a systematic study was conducted on the influence of ultrasonic testing parameters on defect detectability, leading to the development of an ultrasonic inspection procedure for laser welds in medium-thick titanium alloy plates. This research can provide technical support for the non-destructive evaluation of laser welding quality in medium-thick titanium alloys and offer experimental evidence for the refinement of relevant inspection standards. The specific novelty and contribution of this work lie in addressing the ultrasonic inspection needs for the particular combination of titanium alloy, laser welding, and medium thickness. By integrating finite element simulation with experimental validation, this study clarifies the trade-off between penetration capability and background response suppression at different frequencies. This provides a preliminary quantitative basis for transducer frequency selection in this specific context. Furthermore, the practical utility of a dedicated mono-crystal shear wave angle transducer for this application is validated, offering technical references for refining relevant inspection standards and engineering practices.

## 2. Materials and Methods

### 2.1. Theory and Numerical Model

#### 2.1.1. Propagation Characteristics of Ultrasonic Waves in Workpieces

**Figure 1:**
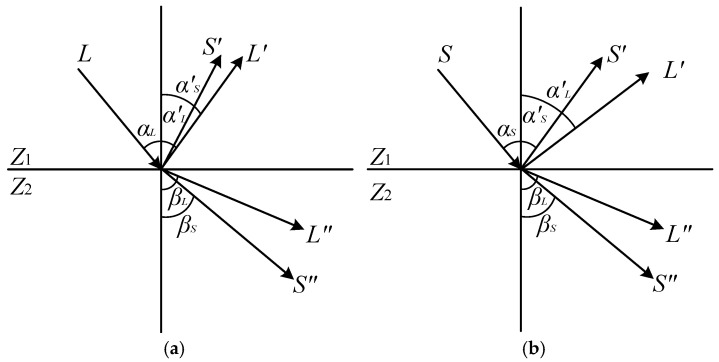
Schematic diagram of oblique wave incidence. (**a**) Longitudinal wave incidence. (**b**) Shear wave incidence. (The vertical line in the figure represents the normal line).

Ultrasonic non-destructive testing technology utilizes various propagation characteristics of ultrasonic waves in objects to measure information such as the dimensions, density, internal defects, and microstructural changes in workpieces made from different materials [21]. Among current ultrasonic testing methods, the pulse-echo method is the most widely used. In the pulse-echo method, when a longitudinal wave is incident on the workpiece interface at different oblique angles, wave mode conversion occurs, generating shear waves, surface waves, or plate waves within the workpiece, which are then utilized for flaw detection. Mode conversion refers to the phenomenon where, in addition to the same type of reflected and refracted waves, different types of reflected and refracted waves are generated when ultrasonic waves are incident obliquely at an interface [22]. As illustrated in Figure 1a, when a longitudinal wave (L) is incident obliquely from medium Z_1_ to medium Z_2_, besides the reflected longitudinal wave (L′) and refracted longitudinal wave (L″), a reflected shear wave (S′) and a refracted shear wave (S″) are also produced. Similarly, Figure 1b depicts the mode conversion that occurs when a shear wave is incident obliquely on an interface. The directions of the various reflected and refracted waves conform to the following laws of reflection and refraction:

(1) $$\left\{\begin{array}{c} \frac{\sin\alpha_{L}}{c_{L1}}=\frac{\sin{\alpha^{\prime }}_{L}}{c_{L1}}=\frac{\sin{\alpha^{\prime }}_{S}}{c_{S1}}=\frac{\sin\beta_{L}}{c_{L2}}=\frac{\sin\beta_{S}}{c_{S2}}\\ \frac{\sin\alpha_{S}}{c_{S1}}=\frac{\sin{\alpha^{\prime }}_{L}}{c_{L1}}=\frac{\sin{\alpha^{\prime }}_{S}}{c_{S1}}=\frac{\sin\beta_{L}}{c_{L2}}=\frac{\sin\beta_{S}}{c_{S2}} \end{array}\right.$$

where $\alpha_{L}$ and $\alpha_{S}$ are the incident angles of the incident longitudinal wave (L) and incident shear wave (S), respectively; ${\alpha^{\prime }}_{L}$ and ${\alpha^{\prime }}_{S}$ are the reflection angles of the reflected longitudinal wave (L′) and reflected shear wave (S′), respectively; $\beta_{L}$ and $\beta_{S}$ are the refraction angles of the refracted longitudinal wave (L″) and refracted shear wave (S″), respectively; and $c_{L1}$, $c_{L2}$, $c_{S1}$, $c_{S2}$ are the corresponding sound velocities of these waves in their respective media. The reflection and refraction laws expressed by Equation (1) are based on the following idealized assumptions: (a) the interface between the two media is an infinitely large planar interface, neglecting the effects of surface roughness and curvature; (b) both media are isotropic linear elastic media, i.e., the sound velocity does not vary with the propagation direction. In practical inspection, titanium alloy weld microstructures exhibit a certain degree of anisotropy; however, as an engineering approximation, the above assumptions still provide guidance with sufficient accuracy.

Since the velocity of longitudinal waves remains constant within the same medium, it follows that ${\alpha^{\prime }}_{L}$ = $\alpha_{L}$. Furthermore, as the velocity of longitudinal waves is greater than that of shear waves within the same medium, we have ${\alpha^{\prime }}_{L}$ > ${\alpha^{\prime }}_{S}$, $\beta_{L}$ > $\beta_{S}$. When $\beta_{L}=90{}^{\circ}$, the corresponding incident angle of the longitudinal wave is defined as the first critical angle $\alpha_{I}$; when $\beta_{S}=90{}^{\circ}$, the corresponding incident angle of the longitudinal wave is defined as the second critical angle $\alpha_{\mathrm{II}}$. From Equation (1), these can be derived as:

(2) $$\left\{\begin{array}{c} \alpha_{I}=\arcsin\frac{c_{L1}}{c_{L2}}\\ \alpha_{\mathrm{II}}=\arcsin\frac{c_{L1}}{c_{S2}} \end{array}\right.$$

In Formula (2), $c_{L1}$, $c_{L2}$, and $c_{S2}$ represent the sound velocities of the incident longitudinal wave L, the refracted longitudinal wave L″, and the refracted shear wave S″ in their respective media. Based on the definitions of $\alpha_{I}$ and $\alpha_{\mathrm{II}}$, when the incident angle $\alpha_{L}$ of the longitudinal wave is greater than the first critical angle $\alpha_{I}$ but less than the second critical angle $\alpha_{\mathrm{II}}$, i.e., $\alpha_{I}<\alpha_{L}<\alpha_{\mathrm{II}}$, it can be ensured that only a shear wave is transmitted from the wedge into the workpiece.

For longitudinal wave inspection, since a normal transducer is typically used with perpendicular incidence, the orientation of the defect directly affects its detectability. When the defect plane is parallel to the inspection surface, the sound beam is incident perpendicularly to the defect surface, resulting in a maximum reflected echo. However, when the defect plane is perpendicular to the inspection surface or at a large oblique angle, the sound beam becomes nearly parallel to the defect plane, leading to a significantly weakened reflected signal; such defects are considered unfavorably oriented and are therefore difficult to detect. In contrast, shear wave inspection employs an angle transducer with oblique incidence. The aforementioned unfavorably oriented defects, particularly surface-breaking cracks, form a large angle with the incident shear wave beam, thereby generating a strong reflected signal and making shear wave inspection highly sensitive to these types of defects. This characteristic enables the widespread application of shear wave inspection in scenarios such as butt welds, steel structural components, and keyways [23].

**Figure 2:**
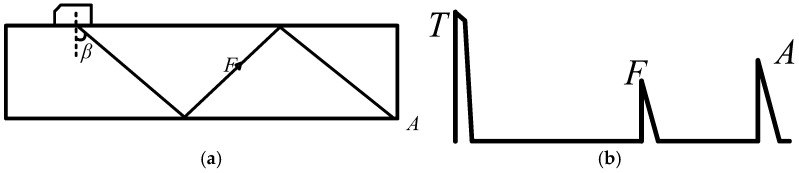
Principle of shear wave inspection. (**a**) Ultrasonic beam propagation path. (**b**) Defect echo waveform.

The principle of shear wave inspection is illustrated in Figure 2. An ultrasonic beam generated by an angle beam transducer is directed into the workpiece at a predetermined refracted angle, as governed by Snell’s law. This beam propagates through the material and reflects from the bottom surface. By moving the transducer, the sound beam effectively scans the entire volume of the weld. If no discontinuity is encountered, the beam energy is largely dissipated or reflected away from the transducer, resulting in only the initial pulse (T) in the received waveform. When the beam interacts with a discontinuity such as a defect, a portion of the energy is reflected back to the transducer, producing a distinct defect echo (F) in the A-scan signal. The time-of-flight and amplitude of this echo provide information about the defect’s location and size. If the beam encounters a corner, such as the weld root or a crack intersecting the bottom surface, a strong corner echo (A) may be generated due to the high corner reflection coefficient. In thin-walled structures, ultrasonic energy may propagate as guided waves (e.g., Lamb waves), and the received signal may include contributions from multiple wave modes and reflections. However, the underlying principles of echo formation from defects and corners remain applicable [24].

#### 2.1.2. Finite Element Simulation

To understand the acoustic field distribution characteristics of the weld specimen and the influence of transducer frequency on detection capability prior to actual inspection, this study employed the Elastic Waves, Explicit interface in COMSOL software (version 6.3) to simulate the propagation process of ultrasonic waves emitted by a shear wave angle transducer within a titanium alloy workpiece. The discontinuous Galerkin finite element method (dG-FEM) with an explicit time integration scheme was adopted to solve the linear elastic wave equation formulated in velocity-strain form, enabling the solution of large-scale transient problems.

The model employs the “Time-Domain Explicit Multiphysics” framework, coupling the piezoelectric effect with the Elastic Waves (Explicit) and Electrostatics interfaces to perform transient modeling of the piezoelectric active region of the ultrasonic transducer. To achieve efficient electro-acoustic energy conversion and progressive acoustic impedance matching within the model, the piezoelectric element is connected to the wedge via a matching layer, with their thicknesses set to half and one-quarter of their respective longitudinal wavelengths. To suppress the ringing of the element after the excitation electrical pulse signal ceases, the piezoelectric element is surrounded by a backing layer. To achieve optimal acoustic energy transmission between the piezoelectric element and the wedge, the acoustic impedance of the matching layer material should be set close to the geometric mean of their acoustic impedances, satisfying the following condition:

(3) $$Z_{\mathrm{matching}}=\sqrt{Z_{\mathrm{transducer}}Z_{\mathrm{wedge}}}$$

where $Z_{\mathrm{matching}}$ is the acoustic impedance of the matching layer, $Z_{\mathrm{transducer}}$ is the acoustic impedance of the piezoelectric element, and $Z_{\mathrm{wedge}}$ is the acoustic impedance of the wedge.

To ensure the accuracy of the acoustic field simulation results, the materials selected for the model were kept consistent with, or close to, those used in the actual fabricated simulated specimens, reference blocks, wedges, and transducers. For instance, the sound velocity for the titanium alloy was set to the experimentally measured value, while the sound velocity for the wedge material was set to its nominal value. Alumina/epoxy and tungsten/epoxy composites are typical materials used for matching layers and backing layers [25]. Table 1 lists the basic properties of the materials employed in this model. Substituting the longitudinal wave velocity of the wedge and the longitudinal and shear wave velocities of the tested workpiece from Table 1 into Equation (2), the calculated results are $\alpha_{I}$ = 26.09° and $\alpha_{\mathrm{II}}$ = 61.04°. In this model, the incident angle of the longitudinal wave $\alpha_{L}$ is set to 30°.

**Figure 3:**
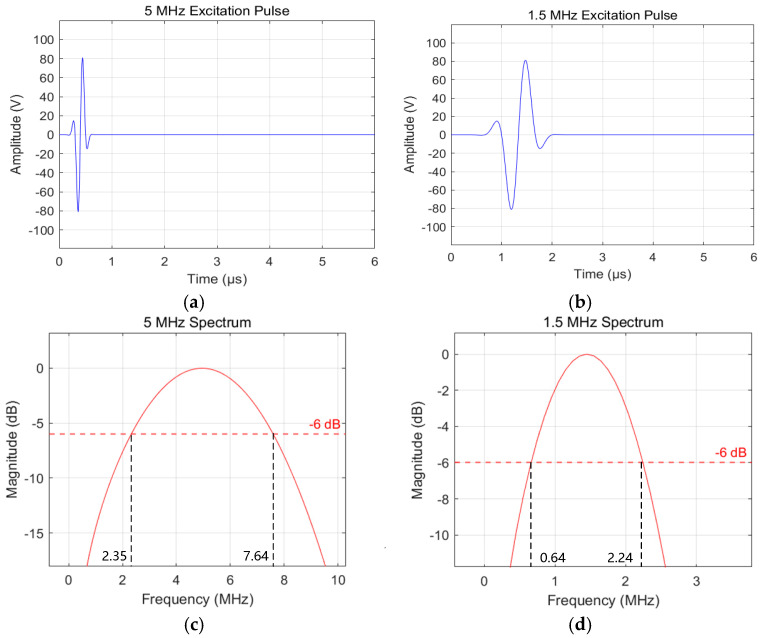
Excitation signal waveform of the voltage source. (**a**) Time-domain waveform of the 5 MHz excitation signal. (**b**) Time-domain waveform of the 1.5 MHz excitation signal. (**c**) Frequency spectrum of the 5 MHz excitation signal. (**d**) Frequency spectrum of the 1.5 MHz excitation signal.

The voltage source is a Gaussian pulse signal with a center frequency of $f_{0}$, expressed as follows:

(4) $$f(t)=100exp(-{\left((t-2T_{0})/(T_{0}/2)\right)}^{2})sin(2\pi f_{0}t)\;0\leq t\leq 10T_{0}$$

where f(t) is the applied excitation electrical signal, $T_{0}$ is the signal period, and $f_{0}$ is the center frequency of the ultrasonic signal. When $T_{0}=200\;\mathrm{ns}$, i.e., $f_{0}=5\;\mathrm{MHz}$, the waveform and frequency spectrum of the excitation signal are shown in Figure 3a and c, respectively. When $f_{0}=1.5\;\mathrm{MHz}$, the waveform and frequency spectrum of the excitation signal are shown in Figure 3b and d, respectively. The input signal amplitudes for both frequencies were kept identical. Reference [26] points out the relationship between detectable defect size and wave frequency: for a defect to be observable, its size must be of the same order of magnitude as the minimum wavelength excited in the medium. From Figure 3b,d, the effective frequency ranges contained in the two excitation signals (calculated by the −6 dB method) are obtained. Subsequently, the minimum wavelength $\lambda_{\min}$ and maximum wavelength $\lambda_{\max}$ in the signals can be calculated using the following formulas:

(5) $$\left\{\begin{array}{c} \lambda_{\min}=\frac{c_{T}}{f_{\max}}\\ \lambda_{\max}=\frac{c_{T}}{f_{\min}} \end{array}\right.$$

In Formula (5), $c_{T}$ is the shear wave velocity of the tested workpiece in the model, $c_{T}$ = 3120 m/s, $f_{\max}$ and $f_{\min}$ are the maximum and minimum effective frequencies contained in the excitation signal, $f_{\max}$ = 7.64 MHz, $f_{\min}$ = 0.64 MHz. The calculated result is $\lambda_{\min}$ = 0.41 mm, $\lambda_{\max}$ = 4.88 mm.

**Figure 4:**
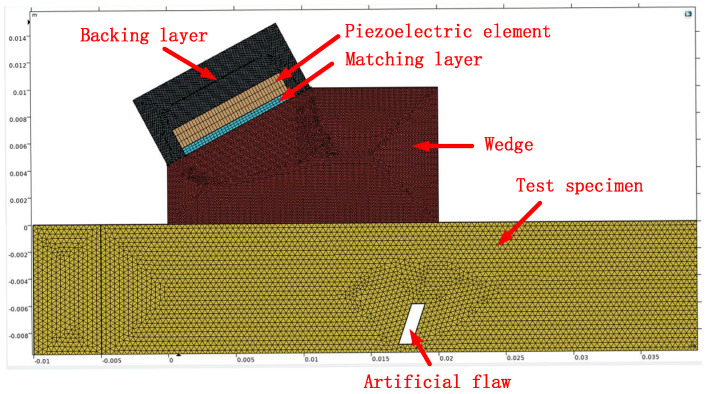
Shear wave inspection model and non-conformal meshing: Backing layer (black), Test specimen (yellow), Wedge (brown), Piezoelectric element (brown), Matching layer (blue), Artificial flaw (white).

To accurately characterize the synergistic effects of the multi-physics fields within the ultrasonic transducer while ensuring computational efficiency, this study employed a geometric assembly and non-conformal meshing strategy to achieve explicit coupled propagation analysis of the piezoelectric–elastic–acoustic fields. Figure 4 illustrates the model geometry and meshing configuration.

To investigate the influence of defect presence and transducer frequency on ultrasonic testing signals, a comparative simulation scheme was established. In COMSOL, a parallelogram-shaped artificial defect with a base length of 1 mm and a height of 3 mm was constructed inside the test specimen using a Boolean difference operation. This defect size was intentionally designed to ensure that both the 1.5 MHz and 5 MHz frequencies theoretically meet the detectable conditions, thereby eliminating the inherent difference in minimum detectable defect size caused by frequency variation and focusing the investigation on the penetration capability and background elastic response level at a thickness of 10 mm. By suppressing or activating this Boolean difference feature, the model could be rapidly switched between the intact model and the defect-containing model, enabling a direct comparison of results with and without the defect. Based on these two geometric configurations, excitation frequencies of 1.5 MHz and 5 MHz were further applied to the piezoelectric element, and the ultrasonic responses under different combinations were calculated. By comparing the defect echo amplitude and background elastic response characteristics, the influence of frequency on the inspection capability for medium-thick titanium alloy laser welds was analyzed.

**Figure 5:**
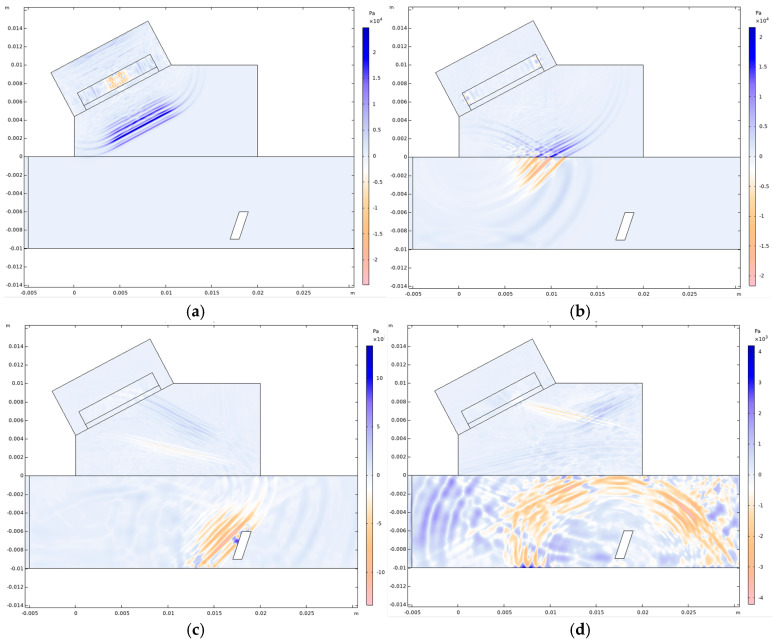
Acoustic field distribution characteristics at $f_{0}$ = 5 MHz (**a**) Waveform at t = 3 μs. (**b**) Waveform at t = 5 μs. (**c**) Waveform at t = 8 μs. (**d**) Waveform at t = 12 μs.

Figure 5 illustrates the propagation process of the signal from the transducer to the simulated defect within the test specimen. Longitudinal waves are shown in blue, and shear waves are shown in orange. The longitudinal wave generated by the transducer travels through the wedge (t = 3 μs). It then impinges on the surface of the test specimen, whereupon the refracted shear wave begins to propagate through it (t = 5 μs) and advances towards the simulated defect (t = 8 μs). Upon striking the defect, the wave is reflected back towards the wedge (t = 12 μs).

**Figure 6:**
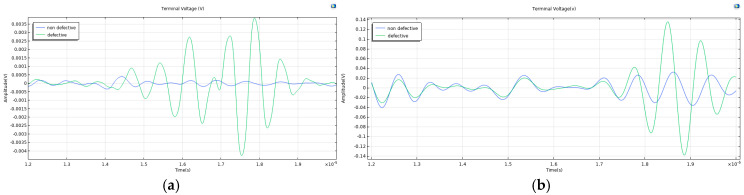
Enlarged view of the voltage signals at the transducer terminals for test specimens with a defect (green) and without a defect (blue). (**a**) $f_{0}$ = 5 MHz. (**b**) $f_{0}$ = 1.5 MHz.

Figure 6 presents a comparison of the voltage signals received by the transducer under conditions with and without a defect in the test specimen, excited by 5 MHz and 1.5 MHz signals, respectively. As can be seen from the figure, compared to the background elastic response in the defect-free state, the defect echo signals in the presence of a defect are clearly discernible for both frequencies. This indicates that both testing frequencies can effectively detect the simulated defect, thereby validating the rationality of the chosen defect size. Although there is a significant difference in the amplitude of the echo signals between the two frequencies, the ratio of the defect echo amplitude to the background elastic response amplitude is found to be of the same order of magnitude. Specifically, at 5 MHz, the background elastic response amplitude in the defect-free state is low (approximately 0.5 mV), while the defect echo amplitude in the presence of a defect is about 3.7 mV. At 1.5 MHz, the background elastic response amplitude in the defect-free state is considerably higher (approximately 30 mV), and the defect echo amplitude reaches 135 mV. This result is consistent with the theory of ultrasonic attenuation—lower-frequency signals experience less attenuation during propagation in a medium and thus yield higher echo amplitudes, confirming that the simulation model correctly captures the fundamental propagation behavior of ultrasonic waves in titanium alloys.

Furthermore, considering that all physical processes are modeled in the linear regime, larger signal amplitudes can be achieved simply by proportionally increasing the excitation voltage. In subsequent experimental testing, the relatively lower defect echo amplitude at 5 MHz can likewise be compensated by appropriately increasing the excitation voltage, an approach that is both feasible and effective in practical engineering. However, the inherent disadvantage of the 1.5 MHz transducer in detecting small-size defects cannot be readily mitigated by simply adjusting the gain. Therefore, it can be anticipated that when the defect size is reduced to the equivalent dimensions used in the experimental part of this study (φ1 mm and φ1.5 mm), the 5 MHz transducer will yield better detection performance.

It should be noted that no artificial noise was added in this simulation; all background elastic responses originate from reflections at the workpiece boundaries and structural features. The above results are intended to reveal the dual influence of frequency on defect echo and background response, rather than to simulate electronic noise or environmental interference in actual testing. Furthermore, several simplifications inherent in the simulation model should be acknowledged. The material is modeled as homogeneous and isotropic, whereas actual titanium alloy weld microstructures are coarse-grained and anisotropic, meaning that a significant practical source of structural noise (scattering at grain boundaries) is not explicitly captured. Additionally, the artificial defect is represented by an idealized geometric void, which simplifies the complex morphology of actual weld defects (e.g., lack of fusion, porosity). The model also assumes a perfectly flat surface, neglecting the effects of weld reinforcement or surface irregularities on transducer coupling. Consequently, this simulation primarily aims to reveal fundamental trends, and further validation through experimental testing remains necessary.

### 2.2. Materials and Experimental Methods

#### 2.2.1. Preparation of Reference Blocks and Specimens

**Figure 7:**
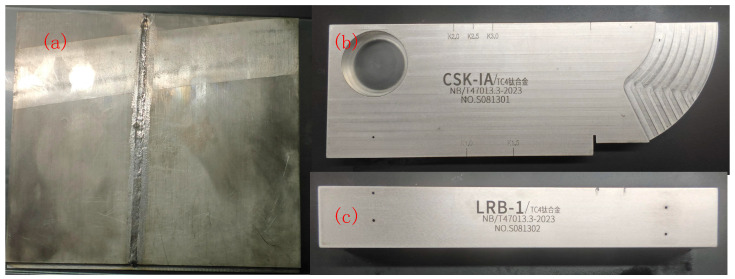
Physical photographs of reference blocks and specimens. The Chinese characters denote the material grade (TC4 titanium alloy). (**a**) Test specimen. (**b**) Titanium alloy CSK-IA calibration block. (**c**) LRB-1 type reference block.

**Figure 8:**
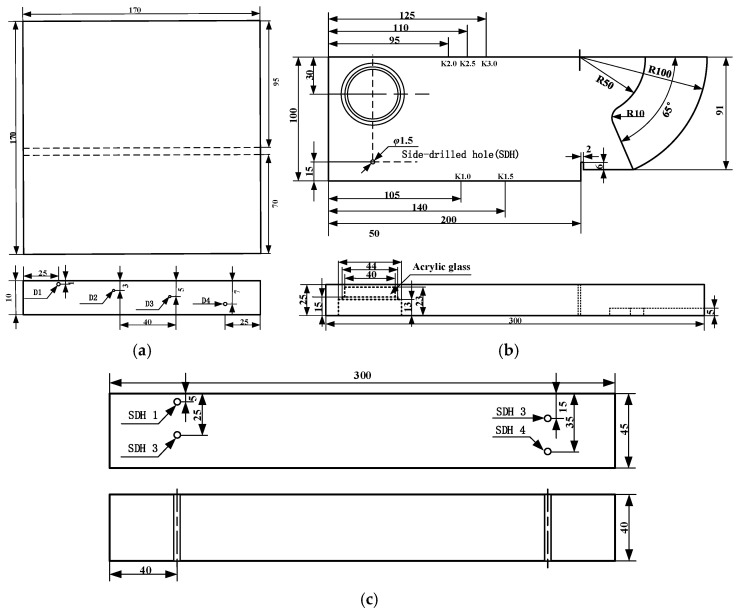
Dimensional drawings of reference blocks and specimens. (**a**) Test specimen, the dash line represents the weld. (**b**) Titanium alloy CSK-IA calibration block. (**c**) LRB-1 type reference block, the dash line indicates the position of the hole.

In the experimental preparation, butt welding was performed on the tested workpiece using an Nd:YAG solid-state laser with the following welding parameters: a laser power of 3 kW, a welding speed of 0.8 m/min, and argon shielding. Concurrently, calibration blocks and dedicated reference blocks suitable for inspecting titanium alloy welds with thicknesses ranging from 8 mm to 40 mm were fabricated in accordance with Annex H of NB/T 47013.3-2023, “Ultrasonic testing methods and quality classification for butt joints of aluminum, aluminum alloy and titanium pressure equipment” [27]. All artificial defects in this study were processed as side-drilled holes (SDHs) via the drilling method. For the welded test specimen, four side-drilled holes were machined on the faying surfaces prior to welding, distributed at different depths along the through-thickness direction of the plate, with their equivalent sizes pre-calibrated as φ1.5 mm, φ1.0 mm, φ1.0 mm, and φ1.5 mm, respectively. In the LRB-1 type reference block used for establishing the distance–amplitude curve, a total of four φ2 mm side-drilled holes were fabricated, with the distances from their centers to the upper surface of the block being 5 mm, 15 mm, 25 mm, and 35 mm, respectively. After drilling, the hole walls were finished to ensure smoothness and freedom from burrs. Figure 7a illustrates the shape of the test specimen and the laser weld joint configuration. Figure 7b shows a photograph of the fabricated titanium alloy CSK-IA calibration block, and Figure 7c shows a photograph of the LRB-1 type reference block. Figure 8a–c detail their respective dimensions, with all units in millimeters. Table 2 specifies the locations and equivalent sizes of the prefabricated artificial defects in the test specimens and reference blocks.

#### 2.2.2. Ultrasonic Testing System

**Figure 9:**
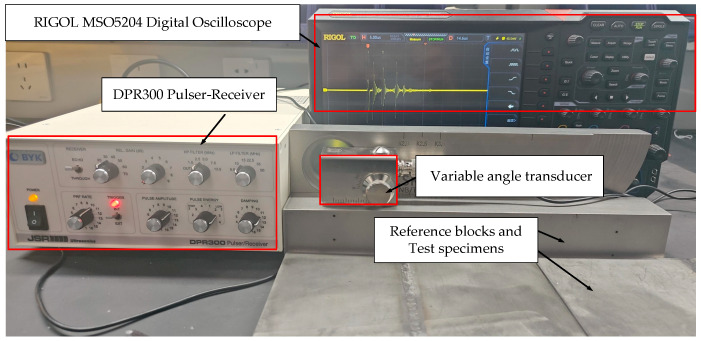
Experimental setup used in this study.

The ultrasonic testing system established in this study is illustrated in Figure 9. It primarily consists of a DPR300 ultrasonic pulser-receiver, a variable angle transducer, an MSO5204 digital oscilloscope, and CSK-IA standard reference blocks/LRB-1 type reference blocks/test specimens. The DPR300 pulser-receiver generates high-voltage electrical pulses to excite the transducer and receives the reflected echo signals from within the specimen. This instrument features a bandwidth of 35 MHz and a receiver gain range of −13 dB to +66 dB, allowing adjustable excitation energy and receiving sensitivity according to inspection requirements. A 5 MHz variable angle transducer (SIUI, Shantou, China) was employed, which allows adjustment of the incident angle via a control knob to generate shear waves with specific refraction angles within the titanium alloy specimen, thereby expanding the applicability to various workpiece geometries. The excitation and echo signals were acquired and displayed in real time using an MSO5204 digital oscilloscope. This oscilloscope has a sampling rate of 8 GSa/s and an analog bandwidth of 200 MHz, enabling precise recording of the time-domain ultrasonic waveforms. The acquired waveform data were exported in CSV format for subsequent signal-to-noise ratio analysis and DAC curve construction.

#### 2.2.3. Experimental Method

**Figure 10:**
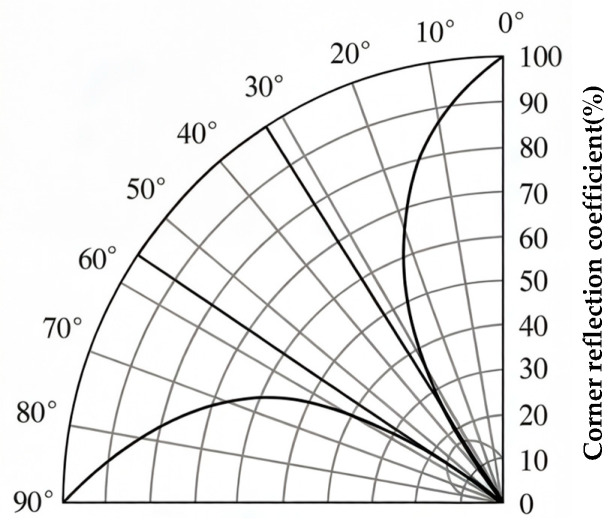
Relationship between corner reflection coefficient and shear wave incident angle.

Given the feature of laser welds having no groove, the selection of the incident angle was based on the corner reflection coefficient curve shown in Figure 10 [28]. It is known from the figure that, theoretically, the corner reflection coefficient for shear waves is minimal when the incident angle $\alpha_{S}$ is near 30° or 60°, and reaches 100% when $\alpha_{S}$ is between 35° and 55°. This behavior is analogous to the detection of lack of penetration defects in grooved welds, where the laser weld root resembles the root face. By continuously adjusting the angle of the transducer within the calculated critical angle interval, it was found that the optimal reflection effect was achieved when the longitudinal wave incident angle $\alpha_{L}$ was 40°. Therefore, this angle was set as the longitudinal wave incident angle of the transducer in the experiment. The corresponding K-value of the transducer was measured to be 1.48, i.e., a shear wave refraction angle $\beta_{S}$ was 56°. All subsequent experimental results presented in this study were obtained using this angle.

**Figure 11:**
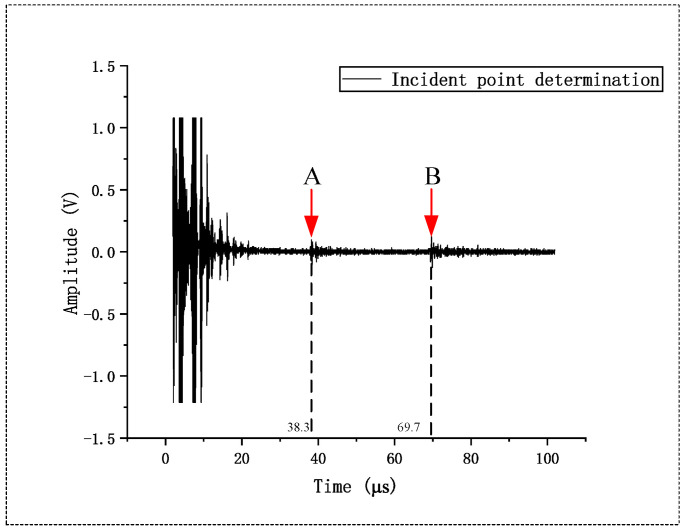
Schematic diagram of incident point determination using the CSK-IA standard reference block. In the figure, point A represents the maximum echo from the R50 mm arc surface; point B represents the maximum echo from the R100 mm arc surface.

System calibration was performed using a CSK-IA standard reference block. The incident point and wedge delay were calibrated using the R50 mm and R100 mm arc surfaces on the block. Figure 11 shows the maximum reflection echoes obtained from the R50 mm and R100 mm arc surfaces. At this point, the position on the side of the transducer corresponding to the scale line on the block marks the incident point of the transducer. Furthermore, the propagation time required for a 100 mm sound path, denoted as $T_{100}$, can be calculated based on the horizontal axis coordinates corresponding to points A and B in Figure 11, using the following formula:

(6) $$T_{100}=T_{B}-T_{A}$$

where $T_{A}$ and $T_{B}$ are the times corresponding to points A and B in Figure 11, respectively, with $T_{A}$ = 38.3 μs, $T_{B}$ = 69.7 μs. Thus, $T_{100}$ = 31.4 μs is obtained. Based on this, the shear wave velocity $c_{S}$ in the reference block used in this study can be calculated according to Equation (6):

(7) $$c_{S}=\frac{s_{100}}{T_{100}}$$

where $s_{100}$ is the sound path length, and $T_{100}$ is the time required to propagate this 100 mm sound path. The calculated result is $c_{S}$ = 3184.7 m/s.

To facilitate subsequent defect localization and sound path calculation, the zero offset of the system needs to be calibrated. Zero offset refers to the time delay from the triggering of the excitation pulse to the moment the ultrasonic wave enters the surface of the test workpiece. This delay directly affects the correspondence between the echo time and the sound path and must be accurately compensated for prior to inspection. The zero offset $t_{0}$ is calculated using the following formula:

(8) $$t_{0}=\frac{T_{A}-(T_{B}-T_{A})}{2}$$

The calculated result is $t_{0}$ = 3.45 μs.

**Figure 12:**
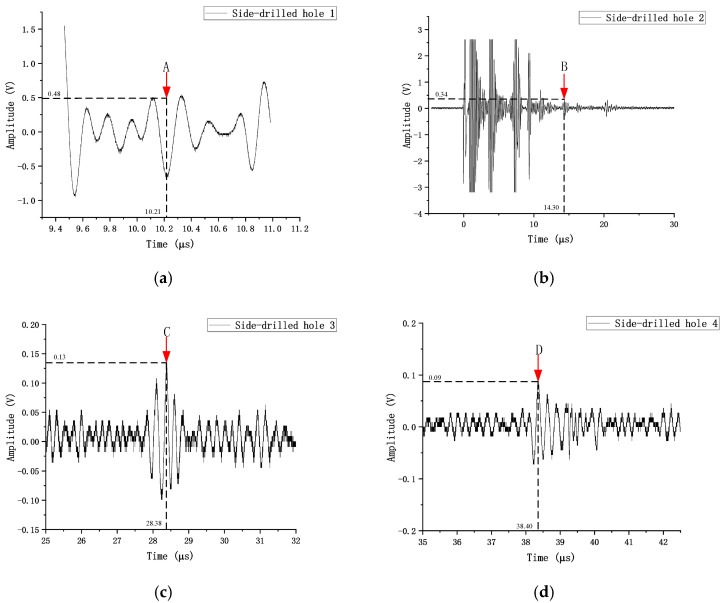
Ultrasonic echoes from φ2 mm side-drilled holes at different depths in the LRB-1 reference block. (**a**) SDH 1. (**b**) SDH 2. (**c**) SDH 3. (**d**) SDH 4.

**Figure 13:**
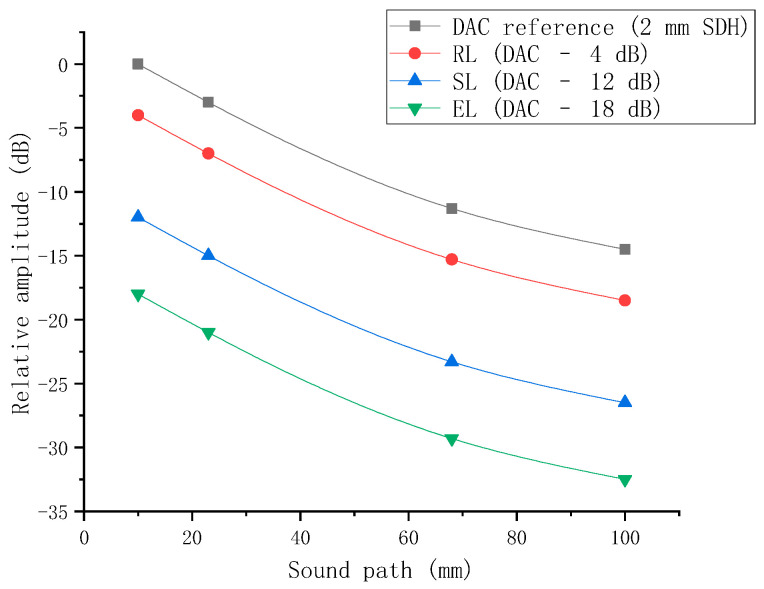
Distance–amplitude (DAC) curve.

The distance–amplitude curve (DAC curve) was constructed using a dedicated LRB-1 type reference block. The transducer was sequentially placed at the positions corresponding to the side-drilled holes on the block diagram, as illustrated in Figure 12. By moving the transducer back and forth, the position yielding the maximum reflected echo was located, and the corresponding parameters on the DPR300 panel were recorded. Using the four measurement points A, B, C, and D shown in the figure, the reference DAC curve was constructed with sound path as the abscissa and relative amplitude (dB) as the ordinate by fitting these points. This curve represents the variation in the reflected wave amplitude from a φ2 mm side-drilled hole as a function of sound path and serves as the basis for subsequent defect quantitative evaluation. According to the sensitivity requirements specified in Annex H of NB/T 47013.3-2023, the evaluation line (EL), sizing line (SL), and rejection line (RL) were obtained by shifting the reference DAC curve downward by 18 dB, 12 dB, and 4 dB, respectively. This yields the complete distance–amplitude curve, as shown in Figure 13.

**Figure 14:**
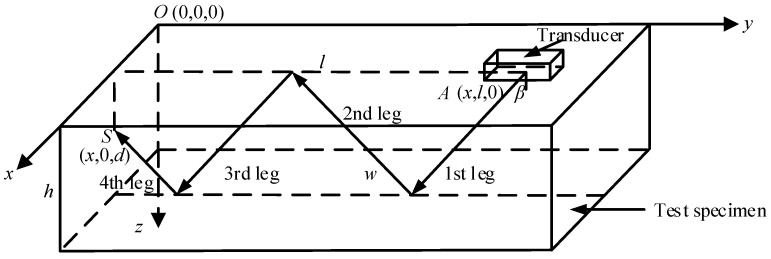
Three-dimensional Cartesian coordinate system for defect localization.

To enable precise spatial localization of detected defects, a three-dimensional Cartesian coordinate system was established, as shown in Figure 14. The origin *O*(0, 0, 0) is set at a corner of the workpiece’s top surface, with the x-axis oriented along the weld direction, the y-axis oriented horizontally perpendicular to the weld, and the z-axis pointing vertically downward into the workpiece to represent depth. The thickness of the specimen is denoted as *h*. The coordinates of the transducer incident point are defined as *A*(*x*, *l*, 0), and the coordinates of the defect point as *S*(*x*, 0, *d*). Here, *x* is the coordinate along the weld direction (known for all four prefabricated defects), *l* is the horizontal projection distance from the transducer incident point to the defect, and *d* is the desired vertical depth of the defect. β represents the shear wave refraction angle. Defect localization is based on the ultrasonic wave propagation time. Assuming the total time of flight for the defect echo measured by the instrument is *t*_D_, and the system zero offset is $t_{0}$, the one-way sound path *w* from the incident point *A* to the defect *S* is calculated using the following formula:

(9) $$w=\frac{c_{S}(t_{D}-2t_{0})}{2}$$

where $c_{S}$ is the shear wave velocity in the workpiece specimen. In shear wave inspection, given the specimen thickness *h* and the shear wave refraction angle β, the formulas for calculating *l* and *d* are as follows:

(10) $$l=w\sin\beta =d^{\prime }\tan\beta$$

(11) $$\left\{\begin{array}{cc} d=w\cos\beta -(n-1)h=d^{\prime }-(n-1)h & n=1,3,5\ldots \\ d=nh-w\cos\beta =nh-d^{\prime } & n=2,4,6\ldots \end{array}\right.$$

where $d^{\prime }$ is the nominal depth of the defect, with $d^{\prime }=w\cos\beta$, where *n* represents the wave mode number *n* = 4 in Figure 14.

## 3. Results and Discussion

**Figure 15:**
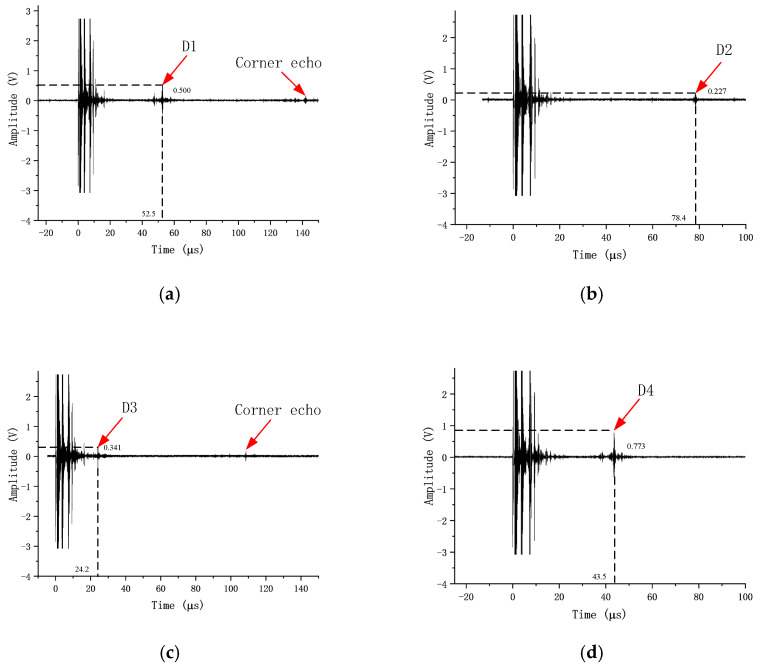
Waveforms of detected defects. (**a**) Defect D1. (**b**) Defect D2. (**c**) Defect D3. (**d**) Defect D4.

It should be noted that manual scanning was adopted in the experimental part of this study, as the test specimens were relatively small, and manual operation was sufficient to meet the inspection requirements. The presence of defects was determined using a W-scanning pattern. A combination of forward–backward and left–right scanning was employed to locate the maximum wave height of the defect for subsequent defect localization and amplitude measurement. At this position, the coordinates of the transducer incident point and the parameters on the DPR300 panel were recorded. All prefabricated defects were successfully detected. Figure 15 shows the echo waveforms corresponding to the four detected defects. From the figure, it can be observed that the overall signal-to-noise ratio (SNR) is satisfactory, and the defect echoes are clearly distinguishable. Calculations yield SNR values of 20.92 dB, 14.06 dB, 16.87 dB, and 18.58 dB for the four defects, with an average value of approximately 17.6 dB, meeting the requirements for quantitative analysis and localization.

The amplitude values *V*_meas_ corresponding to the defect echoes were obtained from the waveforms in Figure 15. Since the gain of the DPR300 during the actual measurements was 45 dB, which is 15 dB higher than the 30 dB reference gain used for constructing the distance–amplitude (DAC) curve, a gain normalization procedure was applied to the amplitude values. The measured amplitudes were converted to equivalent amplitudes *V*_norm_ at 30 dB gain using the voltage attenuation factor 10^−15/20^. Subsequently, using the reference point of the DAC curve (the echo amplitude of 0.48 V from the φ2 mm side-drilled hole at a depth of 5 mm) as the 0 dB reference, the normalized relative wave heights for each defect were calculated as follows:

(12) $$dB=20\log10(\frac{V_{norm}}{0.48})$$

**Figure 16:**
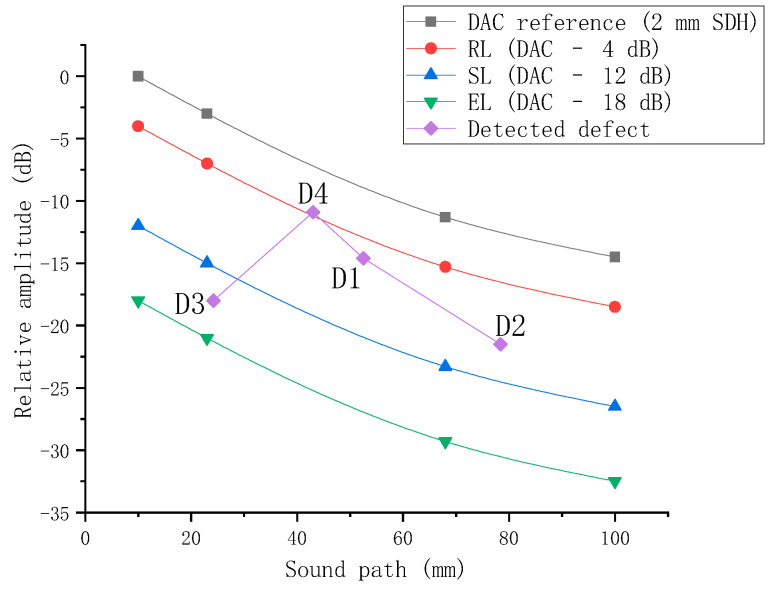
Comparison results of detected defects with the DAC curve.

Following the above processing, the normalized data points for the four defects were obtained. These data points will be directly compared with the DAC curve for defect classification. The results are shown in Figure 16. It can be observed that defects D2 and D3 (with prefabricated defect equivalent sizes of φ1.0 mm) are located near the sizing line (SL), while defects D1 and D4 (with prefabricated defect equivalent sizes of φ1.5 mm) are situated close to the rejection line (RL). These findings are generally consistent with the expected outcomes. Further investigation into precise quantitative methods will be conducted in subsequent studies.

The propagation times *t*_D_ corresponding to the defect echoes were obtained from the waveforms in Figure 15. Substituting $\beta =\beta_{S}=56{}^{\circ}$ and applying Equations (10) and (11), the three-dimensional coordinates of the four defects within the coordinate system shown in Figure 14 were calculated. The results are presented in Table 3.

The localization errors were calculated by comparing the actual depths of the four prefabricated defects with the ultrasonic testing localization results, as presented in Table 4.

The results in Table 4 indicate that the overall localization accuracy of this study meets the requirements for engineering applications (error ≤ 0.5 mm). However, the deviation at the depth of 3 mm slightly exceeds this limit, which may be related to the acoustic field distribution in the near-field zone or the nominal error of the transducer K-value. It is worth noting that in this study, the weld was treated as a zero-thickness cross-section along the y-axis during defect localization. Therefore, the horizontal distance l in Table 3 is not reflected in the final three-dimensional coordinates of the defects. Nevertheless, it can be anticipated that in future research, by recording the specific position of the incident point, more accurate defect localization can be achieved.

It should be noted that the experimental validation in this study was conducted using prefabricated side-drilled holes (SDHs) as artificial defects. While SDHs provide a well-controlled reference for evaluating detection sensitivity and localization accuracy, they differ from actual weld defects in several respects. Realistic defects in laser welds, such as lack of fusion, porosity, and cracks, often exhibit irregular geometries, rough surfaces, and complex orientations, which can influence the reflection characteristics and the resulting echo amplitudes. Nevertheless, the fundamental principle of ultrasonic inspection (which relies on the reflection due to impedance mismatch) remains applicable. Therefore, the core capability of detecting and locating internal discontinuities, as demonstrated with SDHs, provides a strong indicative basis for the inspection of real defects, although the absolute echo amplitudes and the signal-to-noise ratio may vary depending on the specific defect morphology. Future work will focus on validating the proposed method on actual welding defects, including systematic comparisons of detection performance between SDHs and realistic defect types, to further establish the engineering applicability of the technique.

## 4. Conclusions

This study systematically investigated an ultrasonic testing methodology for detecting internal defects in medium-thick titanium alloy laser welds, integrating finite element simulation with experimental validation.

A finite element model for a 5 MHz shear wave angle transducer was developed to analyze ultrasonic propagation characteristics. The simulations revealed a critical insight into the interaction between frequency, defect echo, and background response. It was found that while a lower frequency (1.5 MHz) generates a higher defect echo amplitude, it also induces a significantly higher background response level due to enhanced structural reverberations. In contrast, the higher frequency (5 MHz) exhibits a superior capability to suppress background responses, resulting in a more favorable ratio between the defect signal and background level. This finding underscores that transducer selection should balance penetration capability against background response suppression, rather than solely pursuing high echo amplitude.

An experimental system comprising a DPR300 pulser-receiver and an MSO5204 oscilloscope was established and calibrated. A distance–amplitude curve was constructed following NB/T 47013.3-2023, providing a quantitative basis for defect evaluation. The system successfully detected all prefabricated internal defects, achieving a minimum detectable equivalent size of 1 mm. The experimental results corroborated the simulation trend, confirming the effectiveness of the 5 MHz transducer in practical inspections.

For defect localization, a three-dimensional coordinate system was established based on the transducer incident point and sound path calculations. The localization errors were evaluated, yielding a mean absolute error of 0.438 mm and a root mean square error of 0.462 mm, both meeting the engineering requirement of 0.5 mm accuracy.

In conclusion, this work systematically investigated an ultrasonic testing methodology for medium-thick titanium alloy laser welds, integrating simulation and experiment. The study makes specific contributions in three key areas: (a) addressing the inspection challenge for the specific combination of titanium alloy, laser welding, and medium thickness; (b) clarifying through simulation the trade-off between defect signal amplitude and background elastic response at different frequencies, thereby providing a quantitative rationale for transducer selection; and (c) experimentally validating a dedicated 5 MHz shear wave probe capable of detecting sub-millimeter defects (φ1 mm) with accurate localization (MAE 0.438 mm). The proposed methodology enables effective detection and precise localization. The simulation model offers a tool for understanding how frequency influences the background response, which informs strategies to mitigate structural noise in practice. The experimental findings provide a practical pathway for quality assessment.

## Figures and Tables

**Table 1:** Material Properties of Each Component.

Component	Material	Density (kg/m^3^)	Longitudinal Wave Velocity (m/s)	Shear Wave Velocity (m/s)
Piezoelectric element	PZT-5H	7500	4620	1750
Matching layer	Alumina/Epoxy composite	2280	3400	1920
Backing layer	Tungsten/Epoxy composite	6580	1500	775
Wedge	Polymethyl methacrylate (PMMA)	1190	2730	1430
Test specimen	Titanium alloy (Ti–6Al–4V)	4430	6207	3120

**Table 2:** Parameters of prefabricated artificial defects in test specimens and reference blocks.

Model	Applicable Workpiece Thickness (mm)	Block Thickness (mm)	Defect Depth (mm)	Defect Equivalent Size (mm)
LRB-1	8~40	45	5, 15, 25, 35	φ2.0
Test Specimen	/	10	1, 3, 5, 7	φ1.5, φ1.0, φ1.0, φ1.5

**Table 3:** Parameters of prefabricated defects and experimental results.

Defect No.	Prefabricated Depth (mm)	Defect Equivalent Size (mm)	Measured Vertical Depth *d* (mm)	Measured Horizontal Distance *l* (mm)	Three-Dimensional Coordinates (mm)
D1	1	φ1.5	0.701	20.35	(25, 0, 0.701)
D2	3	φ1.0	3.687	48.31	(65, 0, 3.687)
D3	5	φ1.0	4.648	60.19	(105, 0, 4.648)
D4	7	φ1.5	7.412	94.38	(145, 0, 7.412)

**Table 4:** Defect localization errors.

Mean Absolute Error (MAE)	Root Mean Square Error (RMSE)	Maximum Absolute Error	Average Relative Error (%)
0.438 mm	0.462 mm	0.687 mm	16.4

## Data Availability

The raw data supporting the conclusions of this article will be made available by the authors on request.
